# The timelines for the price and reimbursement authorization in Italy 2018–2020

**DOI:** 10.3389/fmed.2022.1055359

**Published:** 2022-12-21

**Authors:** Valentina Gallo, Eva Alessi, Simona Montilla, Gianluca Altamura, Giuseppe Traversa, Francesco Trotta

**Affiliations:** Italian Medicines Agency, Rome, Italy

**Keywords:** AIFA, medicines access, Price and Reimbursement, time-to-reimbursement, time to access

## Abstract

**Objective:**

This investigation aimed to guarantee the principles of transparency in public administration; to inform citizens about the time to patient access to reimbursed medicines; to assess the duration of the P&R process for the first time in the period 2018–2020; and to evaluate whether and how the SARS-CoV-2 (COVID-19) pandemic affected the P&R activity. This study analyzed the timelines of pricing and reimbursement procedures submitted in Italy by the pharmaceutical marketing authorization holder (MAH) from 2018 to 2020.

**Methods:**

The analysis was run through an AIFA web-based platform that collects data about P&R procedures for each step of the Italian Price and Reimbursement (P&R) procedure, including dates of the Technical Scientific Committee (CTS) and Price and Reimbursement Committee (CPR) meetings from January 2018 to December 2020. On this basis, four indicators were developed relating to the completion time of each stage of the P&R negotiation process and were defined in terms of days. In this regard, descriptive analyses, graphical boxplots, and survival curves (Kaplan–Meier) were carried out, studying these indicators in relation to the typology of pharmaceutical procedures.

**Results:**

Overall, in the period 2018–2020, 57.1% of the 2,445 procedures entered were represented by the Off-patent pharmaceuticals procedures (generics, biosimilars, copies, and/or parallel trade). In 2020, the overall process duration for Off-patent pharmaceuticals procedures was equal to 129.8 average days [95% CI: (122.3–137.2)], with a median value of 108.0, whereas for In-patent pharmaceuticals procedures, it was equal to 283.1 average days [95% CI: (267.8–298.5)], with a median value of 284.0. Over time, the trend of the entire duration of the P&R process tended to decrease. In terms of estimated timing for the conclusion of each stage of the P&R negotiation process, the difference between Off-patent and In-patent pharmaceutical procedures was statistically significant by the Log-Rank test.

**Discussion and conclusion:**

This is the first study to examine the time of the P&R process in Italy, from MAH submission to the publication of the final decision in the Italian Official Journal. The time span considered is 3 years, including the first year of the COVID-19 pandemic. Compared to European average times, in Italy, the time necessary for evaluation, authorization for reimbursement, and definition of the price of a medicine can be considered satisfactory.

## 1. Introduction

In recent years, scientific and technological progress produced new medicines and therapies, modifying the natural history of severe diseases, which are potentially curative or targeted for rare diseases. As an emergent consequence, pressure for early and timely access to new therapies is significant for both patients and healthcare systems.

Several comparative studies measured time to access in Europe and other countries, but conclusive results in terms of availability and affordability are difficult to be drawn, given the differences in the regulatory and healthcare systems (1–3).

Cross-country comparison of the approval times has been presented in several articles. Many studies have analyzed Canada's timing, both individually (4) and in relation to other nations (5). One study observed that the median time to approval of new drugs in Canada decreased considerably in the mid-1990s, although it continued to be longer than in countries such as Australia, Sweden, the United Kingdom, and the United States. This cross-country comparison was subsequently reanalyzed for the period 1996–1998 (6), and it was observed that marketing approval times were similar in Canada and Australia (median of 518 and 526 days, respectively). However, both countries had significantly longer approval times than Sweden (median of 371 days), the UK (median of 308 days), and the USA (median of 369 days). These results ensured that Canadian drug approval times were continuously monitored. Other studies (7) have also observed that approval times for new drugs are generally often longer in Canada than in the United States. However, such shorter approval times could reportedly lead to unsafe drugs entering the market. Therefore, it does not seem that approving a drug in a short period of time is a guarantee of quality.

Approval times must also be distinguished based on the type of drug, as will be shown in this article and as detailed in articles already present in the literature (8), which, for example, have treated generics separately.

A recent study about the availability of innovative medicines and the time to patient access in 34 countries (24 EU and 10 non-EU) measured the patients' Waiting to Access Innovative Therapies (W.A.I.T.) indicator. The indicator showed that for medicines approved by EMA between 2016 and 2019, the median time from EU marketing authorization to patient access ranged from 120 days in Germany to 883 days in Romania, and the European Average time was 504 days (mean %) (9).

Focusing on the European Union (UE), the great majority of new medicines (and their generics and biosimilars) are authorized *via* a centralized procedure, managed by the European Medicines Agency (EMA) and followed by a decision of the European Commission. This procedure allows applicants to obtain a marketing authorization valid throughout the entire EU and countries of the European Economic Area (Iceland, Liechtenstein, and Norway). Even though, once the EU marketing authorization has been granted, the pharmaceutical can in principle be marketed, patient access to new medicines is limited by the reimbursement status. Given that decision about pricing and reimbursement is a national and regional competence, its processes, frameworks, and timelines vary profoundly across EU healthcare systems/countries.

The Transparency Directive set out the time frame within which pricing and reimbursement decisions should be completed, that is 180 and 90 days for new pharmaceuticals and generics, respectively. However, these time limits may not be respected for reasons that are not entirely dependent on the negotiation procedure: P&R application delay by the marketing authorization holder (MAH), clock-stop periods to present additional information, and delay in the publication of the decision in the official journal (10).

In addition, the entire duration of the P&R process can be influenced by the complexity of the assessment and the need to guarantee optimal interaction with MAHs during the negotiation process, with several hearings to discuss the most controversial elements of the dossier. Finally, the MAH may opt for long delays or non-launches instead of accepting a relatively low price due to international price referencing (lower prices in some Member States may influence prices in others) and/or parallel trade (11–13).

We conducted an analysis of the time required by the different steps of the pricing and reimbursement process in Italy: from the application for a P&R process to the administrative verification to the subsequent involvement of the AIFA—Advisory Committees (the Scientific Technical Committee—CTS and Price and Reimbursement Committee—CPR), until the final decision, and the publication in the Italian Official Journal. The analysis was conducted with several aims:

- To guarantee the principle of transparency of the Public Administration;
- To inform citizens about the time to patient access to reimbursed medicines;
- To assess the duration of the P&R process for the first time in the period 2018–2020;
- To evaluate whether and how the COVID-19 pandemic affected P&R activity.

In all these articles, the time indicator that was generally commented on and considered the most consistent was the median.

## 2. Methods

### 2.1. The pricing and reimbursement process for reimbursable medicines

The criteria and parameters to be taken into account in the HTA assessment and negotiation process have been originally defined by the Interministerial Committee for Economic Planning (CIPE), Resolution no. 3 of 1 February 2001. The main criteria utilized are the burden of disease, the place in therapy and the availability of alternative treatments, the risk–benefit profile, the therapeutic added value, and the cost-efficacy and impact on the NHS budget. Recently, an Interministerial Decree dated August 2020 established new criteria for pricing and reimbursement to be applied by 1 March 2021 (13, 14).

**Figure 1:**
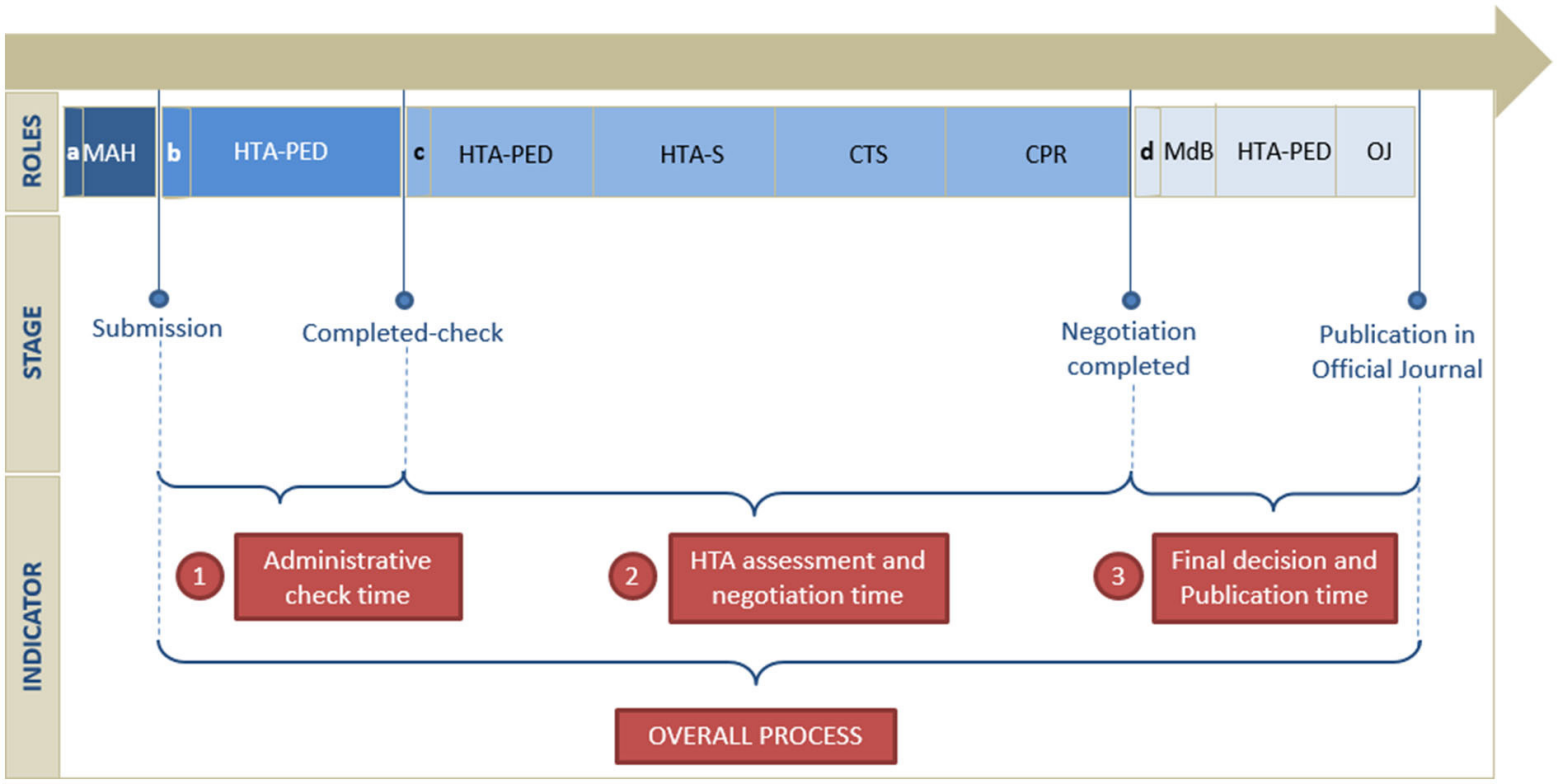
Timeline of the pricing and reimbursement process.

The pricing and reimbursement (P&R) negotiation process occur in four stages, as described in Figure 1:

a) The MAH applies for pricing and reimbursement by submitting a dossier to AIFA.
b) The request is evaluated in terms of administrative completeness (administrative check).
c) AIFA's HTA and Pharmaceutical Economy Division (HTA-PED) and its Secretariat (HTA-S) release a draft assessment to be evaluated by the Scientific Technical Committee (CTS), which issues a binding opinion on the therapeutic value of the medicinal product by defining the place in therapy, on the supply regime, and the degree of innovation. AIFA's Pricing and Reimbursement Committee (CPR) proposes a negotiation agreement to the MAH, where the price and any elements of conditional reimbursement (Managed Entry Agreements—MEA, including monitoring through AIFA's Registries system) are reported, and, when necessary, convenes the MAH for negotiation.
d) The results of the negotiation procedure are submitted to the Management of the Board for the final decision and the procedure is concluded by publication in the Official Journal of the Italian Republic (G.U.).

If case of absence of agreement on the reimbursement scheme and of failure of the negotiation process, the pharmaceutical is classified as non-reimbursable and listed in Class C. Consequently, the price of the product is freely set by the MAH.

### 2.2. The retrospective analysis and the indicators

A retrospective analysis of the P&R negotiation procedures submitted from January 2018 to December 2020 has been conducted. The analysis was run by retrieving data from a web-based platform (NPR system) on October 2021. The NPR system allows the MAHs to electronically apply for P&R negotiation by submitting the dossier and other mandatory documents; tracking and monitoring the process status during the different stages of the negotiation procedure both by the MAHs and AIFA; and collecting information about the typology of negotiation (e.g., new chemical entity, orphan drug, and generic) and administrative data.

In the analysis, four indicators have been developed with the aim of measuring the time duration of each stage of the P&R negotiation process and the entire process. Each indicator is defined as “the number of days obtained between the differences of two dates of the specific step of the process and the entire process,” which are as follows:

- The “Administrative check time” is the time in days elapsed between the completion of the submission date by the MAH and the administrative completed-check step;
- The “HTA assessment and negotiation time” is the time in days elapsed between the date of the completed administrative check and one of the dates indicating the completion of the assessment and of the negotiation process (Management Board meeting for the final decision, date of the non-reached agreement, and date of contract agreement when negotiation is not performed/necessary);
- The “Final decision and Publication time” is the time in days elapsed between the completion of the HTA assessment and negotiation process and the final decision and the publication in the Italian Official Journal;
- The “Overall Process” is the time in days elapsed between the submission date by the MAH and the final decision and publication in the Italian Official Journal.

### 2.3. Statistical analysis

The relationship between the number of procedures, in numbers (*n*) and frequencies (%), time, and typology of negotiation have been evaluated in a contingency matrix (Table 1). The absolute and percentage frequencies of procedures, stratified by the first level of ATC and typology of indications per year, are shown in Supplementary material.

**Table 1:** Distribution of procedures by year and typology of the negotiation procedure.

**Typology of negotiation procedure, per year (frequency** ***n*****, and %)**								
**Typology of negotiation procedure**	**2018**		**2019**		**2020**		**2018–2020**	
	***n***	**%**	***n***	**%**	***n***	**%**	***n***	**% (desc. ord.)**
Off-patent pharmaceuticals procedures (generics, biosimilars, copies and/or parallel trade)	589	62.9%	389	57.5%	418	50.3%	1.396	**57.1%**
Variation of packages, dosage units number, pharmaceutical formulation or device (line extension)	116	12.4%	90	13.3%	96	11.6%	302	**12.4%**
Extension of therapeutic indications/posology	96	10.2%	80	11.8%	114	13.7%	290	**11.9%**
Price and/or reimbursement renegotiation	36	3.8%	41	6.1%	93	11.2%	170	**7.0%**
New active ingredient(s)	30	3.2%	36	5.3%	39	4.7%	105	**4.3%**
Dosage unit variation	22	2.3%	16	2.4%	24	2.9%	62	**2.5%**
Orphan medicines	18	1.9%	11	1.6%	19	2.3%	48	**2.0%**
Other (e.g., market shortages, etc.)	13	1.4%	8	1.2%	5	0.6%	26	**1.1%**
Association of known active ingredients	5	0.5%	3	0.4%	17	2.0%	25	**1.0%**
Reimbursement reclassification	12	1.3%	3	0.4%	6	0.7%	21	**0.9%**
Total	**937**	**100.0%**	**677**	**100.0%**	**831**	**100.0%**	**2.445**	**100.0%**
Total In-patent pharmaceuticals procedures (excluding generics, biosimilars, copies and/or parallel trade)	**348**	**37.1%**	**288**	**42.5%**	**413**	**49.7%**	**1.049**	**42.9%**

The meaning of the bold values indicate the total.

The meaning of the color shade to show the total differentiated by In-patent and Off-patent, that is the principal analysis showed in the our article.

To better capture the differences between the various typologies of the negotiation procedure, two categories of procedures have been built: the “Off-patent pharmaceuticals procedures” and the “In-patent pharmaceuticals procedures,” which includes all the typologies of negotiation described in Table 1, except for procedures regarding generics, biosimilars, copies, and/or parallel trade.

Some descriptive statistics are presented in Table 2, distinguishing between “Off-patent” or “In-patent” pharmaceutical procedures: mean, median, first quartile (Q1), third quartile (Q3), the interquartile difference (IQR), range, and 95% two-sided confidence interval. Specifically, confidence intervals have been built assuming that the sample follows a standard normal distribution.

**Table 2:** Time in days indicators in the period 2018–2020, stratifying the procedure for Off-patent and In-patent pharmaceuticals.

**Time indicators**	**Period**	**% procedures completed**	**Off-patent pharmaceutical procedures**								**In-patent pharmaceutical procedures**							
			***N***	**Mean**	**95% CI (inf-sup)**	**Median**	**Q1**	**Q3**	**IQR (Q3-Q1)**	**Range (min-max)**	***N***	**Mean**	**95% CI (inf-sup)**	**Median**	**Q1**	**Q3**	**IQR (Q3-Q1)**	**Range (min-max)**
Administrative check time	2018–2020	2,445/2,445 (100%)	1,396	8.8	(8.0–9.5)	6.0	2.0	10.0	8.0	(0.0–249.0)	1,049	11.8	(10.5–13.2)	5.0	2.0	12.0	10.0	(0.0–304.0)
HTA assessment and negotiation time		2,266/2,445 (93%)	1,360	94.8	(90.4–99.2)	78.0	49.0	111.0	62.0	(15.0–1.098.0)	906	264.4	(253.9–275)	238.5	143.0	351.0	208.0	(23.0–1.007.0)
Final decision and Publication time		1,715/2,445 (70%)	1,060	65.7	(61.2–70.1)	43.0	33.0	70.0	37.0	(11.0–743.0)	655	66.9	(62.1–71.7)	48.0	35.0	78.0	43.0	(2.0–690.0)
Overall xprocess	2018–2020	1,991/2,445 (81%)	1,247	157.6	(151.6–163.7)	134.0	95.0	184.0	89.0	(28.0–1,173.0)	744	340.0	(327.8–352.3)	307.0	220.5	425.0	204.5	(24.0–1,052.0)
	2018	775/937 (83%)	505	166.7	(157.7–175.7)	146.0	116.0	190.0	74.0	(42.0–1,173.0)	270	390.6	(367.2–413.9)	348.0	244.0	497.0	253.0	(84.0–1,052.0)
	2019	613/677 (91%)	364	173.9	(159.8–188.1)	137.0	95.5	185.5	90.0	(47.0–880.0)	249	336.7	(316.4–357)	321.0	219.0	424.0	205.0	(54.0–962.0)
	2020	603/831 (73%)	378	129.8	(122.3–137.2)	108.0	79.0	162.0	83.0	(28.0–504.0)	225	283.1	(267.8–298.5)	284.0	203.0	367.0	164.0	(24.0–593.0)

The meaning of the color shade to show the total differentiated by In-patent and Off-patent, that is the principal analysis showed in the our article.

The estimate of the indicators has been calculated on a sample of procedures for which the start and end dates of the respective observation period were present (“% procedures completed”): it is given by the percentage ratio between the number of procedures analyzed and concluded on the total of procedures submitted in the NPR system. The remaining percentage represents the procedures that are still in progress or, for some technical reasons, do not have the dates that identify the stage of the P&R negotiation process.

For each indicator, boxplots are presented for “Off-patent” and “In-patent” pharmaceuticals per year (Figure 3). The representation for all typologies of negotiation for the year 2020 can be found in Figure 4.

Considering the duration of each P&R negotiation procedure, from submission to final decision and publication in the Official Journal, Kaplan–Meier curves have been produced for each indicator. Three different approaches have been utilized: (A) all procedures grouped in the period (January 2018–December 2020); (B) all procedures in the period considered and differentiated for “In patent” and “Off-patent” procedures; (C) and all procedures for In-patent pharmaceuticals distributed per year (Figure 5). The Kaplan–Meier curves were compared through the Log-Rank test.

## 3. Results

**Figure 2:**
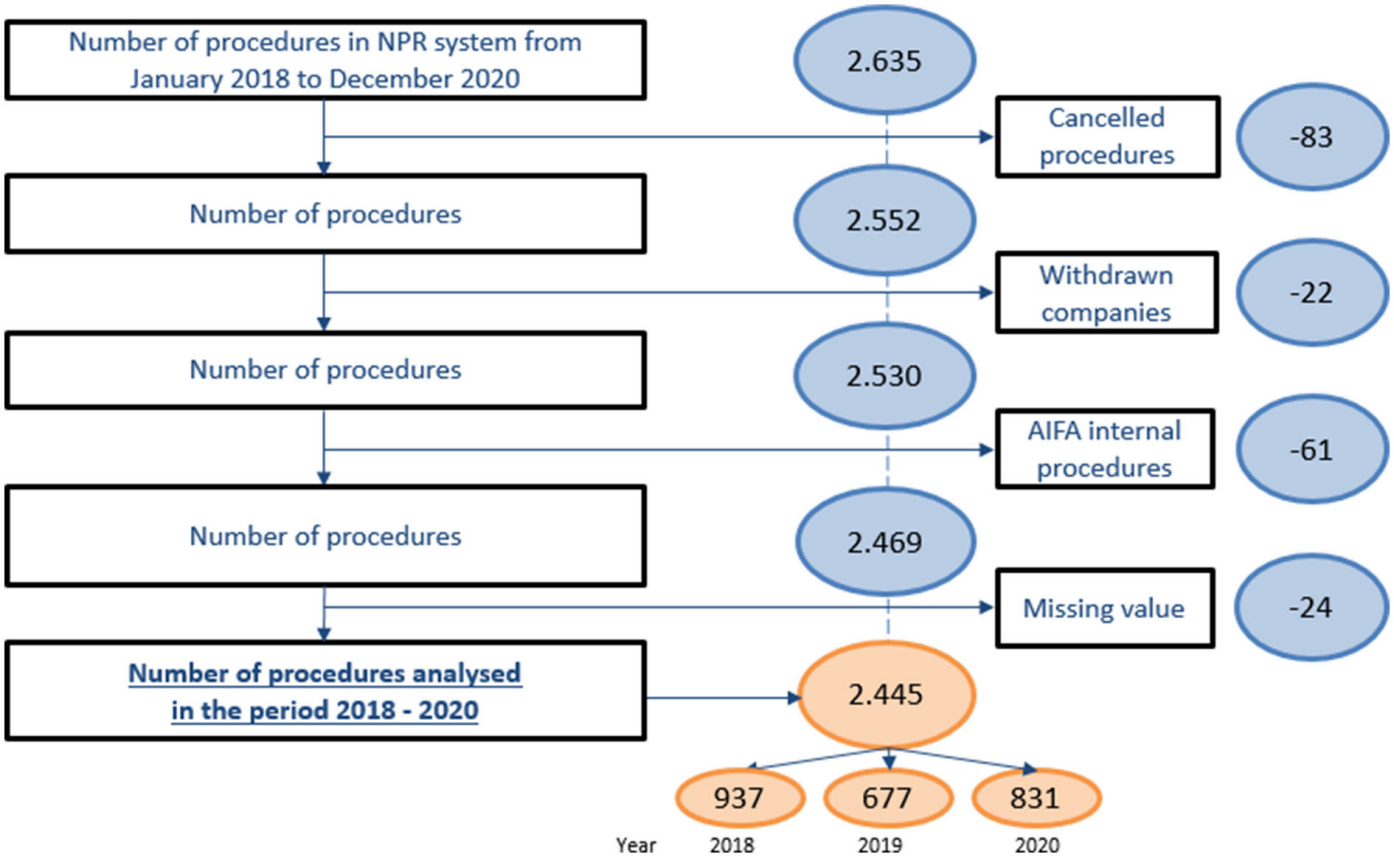
Data set analysis.

The analysis was conducted on 2,445 procedures (Table 1, Figure 2), by excluding the following from the 2,635 procedures submitted in the period considered: 83 canceled, 22 withdrawn by MAHs, and 61 AIFA internal procedures. Moreover, due to missing data concerning the negotiation typology, 24 procedures were further excluded from the data set, of which seven were in 2019 and 17 in 2020.

The number of P&R procedures submitted by the MAH and analyzed per year is higher in 2018 with 937 procedures, followed by a decrease in 2019 with 677 procedures, and a new increase in 2020 with 831 procedures.

Regarding the typology of P&R negotiation procedures, the trend of distribution per year is the same as observed for the overall procedures, with the highest number of submitted procedures per typology found in 2018.

Furthermore, the majority of procedures in the period considered related to generics, biosimilars, copies, and/or parallel trade (1396, 57.1%), followed by line extensions (302, 12.4%) and extension of therapeutic indications (290, 11.9%). For the remaining procedures, those related to orphan drugs (48, 2.0%) remained stable over the period, while those concerning therapeutic indications/posology extension (290, 11.9%), price and/or reimbursement renegotiation (170, 7.0%), and pharmaceuticals based on the new active ingredient(s) (105, 4.3%) increased over the period.

Table 2 shows the main position indices of frequency distributions of the four indicators expressed in the number of days, distinguishing between “Off-patent” and “In-patent” procedures.

### 3.1. The “administrative check time” indicator

**Figure 3:**
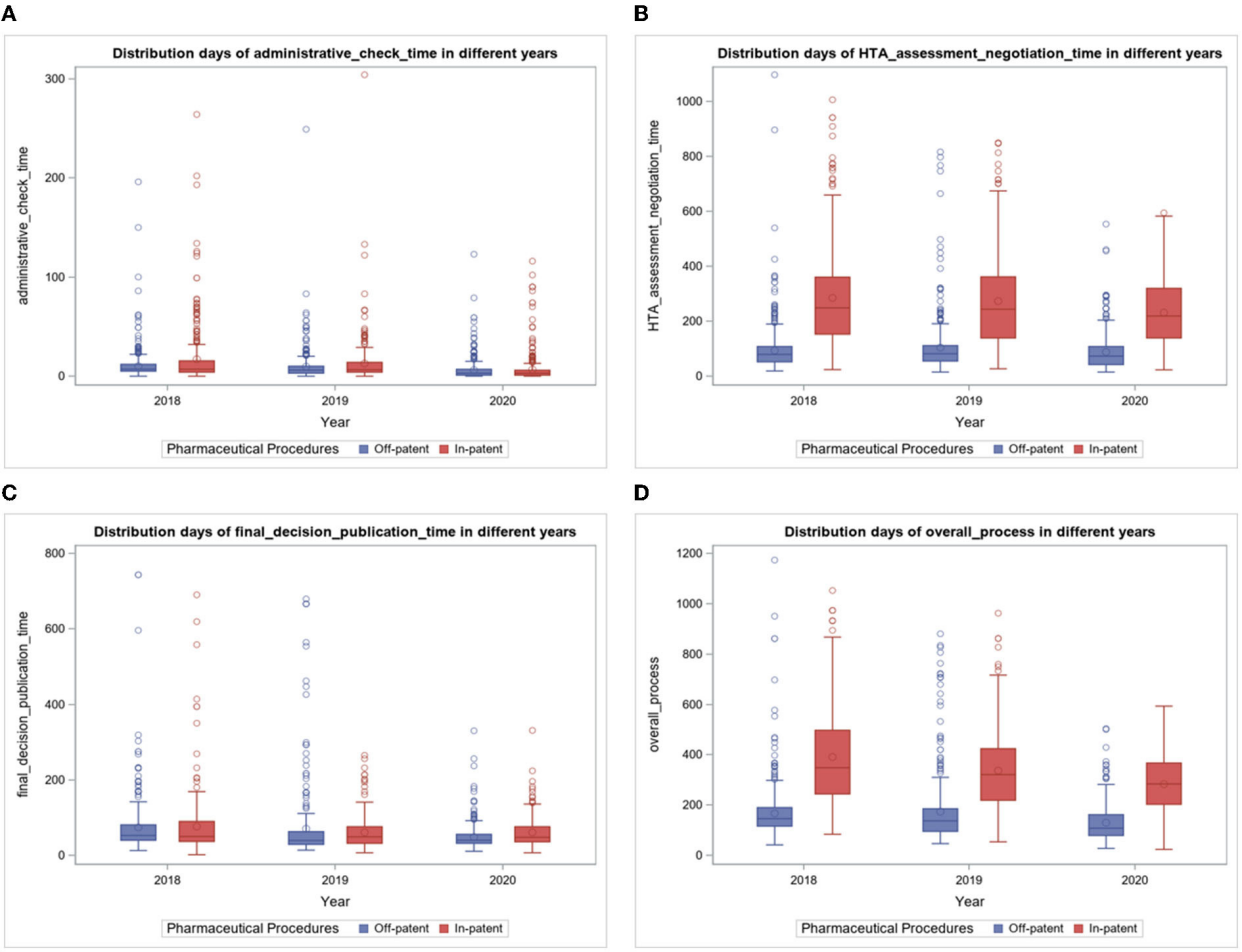
**(A–D)** Boxplots of time days indicators per year.

The “administrative check time” indicator decreased over the years, reaching about 6 days in 2020, both for the procedure concerning Off-patent and In-patent pharmaceuticals (Table 2). The average administrative check time in the period considered is equal to 8.8 for Off-patent procedures and 11.8 for In-patent procedures out of 2,445 procedures entered in the period considered (100% procedures concluded). In the boxplots of Figure 3, it is noted that the indicator slightly decreased in the period considered.

### 3.2. The “HTA assessment and negotiation time” indicator

In the period considered (Table 2), the average days were calculated on 2,266 procedures entered from 2018 to 2020 (at the date of extraction, 93% of the submitted procedures were concluded), resulting in 94.8 days for Off-patent and 264.4 days for In-patent pharmaceuticals procedures. In particular (Figure 3), in 2018, the average was 93.3 days for Off-patent procedures and 285.1 days for In-patent procedures. In 2019, the average HTA assessment and negotiation time was 104.0 days for Off-patent procedures and 273.5 days for In-patent procedures. In 2020, the average HTA assessment and negotiation time was 88.1 days for Off-patent procedures and 231.5 days for In-patent procedures. The indicator for In-patent procedures decreased in the period considered.

**Figure 4:**
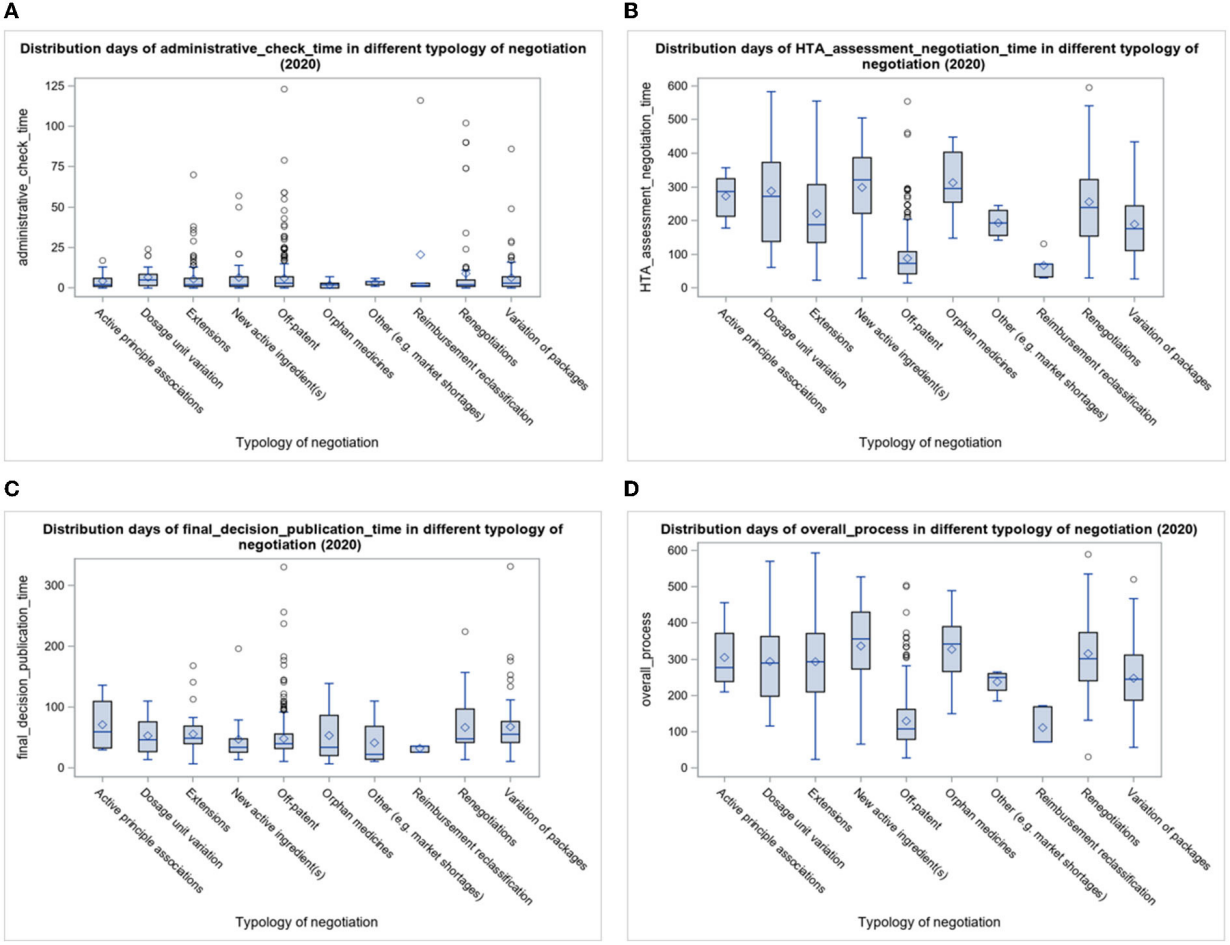
**(A–D)** Boxplots of time days indicators by typology of negotiation.

The indicator is reported by boxplots of the typology of negotiation in Figure 4. Notably, in 2020, fewer days were needed for Off-patent pharmaceuticals procedures (88.1 average days), which is the kind of negotiation with a greater number of procedures concluded. Reimbursement reclassification procedures required about 67.2 average days. More days (on average over half a year) were needed for (in ascending order) P&R procedures concerning the combination of known active ingredients, dosage unit variation, new active ingredient(s), and orphan medicines. The duration of the other typologies of HTA assessment and negotiations procedures is included in the intermediate typology times of typology between the above-mentioned negotiation typology.

### 3.3. The “final decision and publication time” indicator

In the period considered (Table 2), the average days were calculated for 1,715 procedures entered from 2018 to 2020 (at the date of extraction, 70% of the submitted procedures were concluded), resulting in 65.7 days for Off-patent procedures and 66.9 days for In-patent procedures. In particular (Figure 3), in 2018, the average final decision and publication time was 74.3 days for Off-patent procedures and 76.6 days for In-patent procedures. In 2019, the average Final decision and Publication time was 70.7 days for Off-patent procedures and 61.1 days for In-patent procedures. Finally, in 2020, the average final decision and publication time was 48.4 days for Off-patent procedures and 61.0 days for In-patent procedures. In the boxplots of Figure 3, it is noted that the indicator is stable over time with some slight decreases.

### 3.4. The “overall process” indicator

This is the synthetic indication of the entire negotiation process, and in the period considered (Table 2), the average days were calculated on 1,991 procedures entered from 2018 to 2020 (at the date of extraction, 81% of the submitted procedures were concluded), resulting in 157.6 days for Off-patent procedures and 340.0 days for In-patent procedures. The median value drops to 134.0 for Off-patent procedures and 307.0 for In-patent procedures, and this is motivated by the presence of particular extreme cases that produce mean values higher than the median.

In particular (Figure 3), in 2018, the average overall process time was 166.7 days for Off-patent procedures and 390.6 days for In-patent procedures. In 2019, the average overall process time was 173.9 days for Off-patent procedures and 336.7 days for In-patent procedures. In 2020, the average overall process time was 129.8 days for Off-patent procedures and 283.1 days for In-patent procedures. The trend of the entire duration of the P&R process tends to decrease over years.

### 3.5. Box plot and Kaplan–Meier curves

The administrative check time is the shortest phase of the negotiation process, while the HTA assessment and negotiation time is the longest. The final decision and publication time are in the middle.

Regarding the HTA assessment and negotiation time indicator, 50% of procedures in the period lasted approximately 78.0 days for Off-patent procedures and 238.5 days for In-patent procedures. A total of 25% of procedures in the period lasted 49.0 days for Off-patent procedures and 143.0 days for In-patent procedures and 75% of procedures lasted 111.0 days for Off-patent procedures and 351.0 days for In-patent procedures.

In summary, for the second indicator, there is a bigger difference between In-patent and Off-patent pharmaceutical procedures, and this is due to the presence of more negotiation typologies regarding In-patent pharmaceuticals that need several days of negotiation.

In addition, in the In-patent partition, there is more variability compared with the Off-patent one, as can be seen from the distance between the first and third quartiles (IQR) in Table 2. Box plots in Figure 3 represent the time distribution for the years between the two partitions of pharmaceutical procedures (Off-patent and In-patent): the IQR in the In-patent partition is bigger than the Off-patent one.

However, the IQRs related to Off-patent pharmaceuticals procedures remain stable in the period 2018–2020, while for In-patent procedures, they appear to be less stable, that is, the variability of the negotiation process of the procedures in the four indicators is fairly constant (slightly decreasing) over the years.

Mean and median administrative check times are closer than the final decision and publication time and the HTA assessment and negotiation time indicators. In these latter indicators, the median is much lower than the average, due to a greater number of procedures with high completion times. Additionally, distribution is not homogeneous.

In Figure 3, boxplots are useful because the main aspects of the frequency distributions related to the four indicators are highlighted per year. The boxplots of the overall process time indicator show graphically the numbers seen in Table 2.

The variability between Off-patent procedures and In-patent procedures in the other two indicators, administrative check time **(A)** and final decision and publication time **(C)**, is lower than that between HTA assessment and negotiation **(B)** and final decision and publication time indicators **(D)**, as shown in Table 2.

The trends of the administrative check time and final decision and publication time indicators are quite stable over time. Conversely, the HTA assessment and negotiation time and overall process time indicators **(D)** are slightly decreasing in the period considered.

In the second and fourth boxplots [**(B)** and **(D)**] of Figure 4, the “HTA assessment and negotiation” and “overall process” time indicators are shown in the year 2020: “Off-patent” and “Reimbursement reclassification” are the typologies of negotiation for which fewer processing days are required, compared with the others that are longer and more variable. For combinations of active ingredients, dosage unit variation, extensions, new active ingredient(s), orphan medicines, and renegotiations, there are more procedures that take more time to be completed.

Instead, for the Administrative check time indicator **(A)**, the distribution of the boxplot is concentrated in low time values and many outliers for extensions, Off-patent and Renegotiations. For the Final decision and Publication time indicator **(C)** too, more procedures are under 100 days in the year 2020, mainly Off-patent ones, and the IQR is contained.

**Figure 5:**
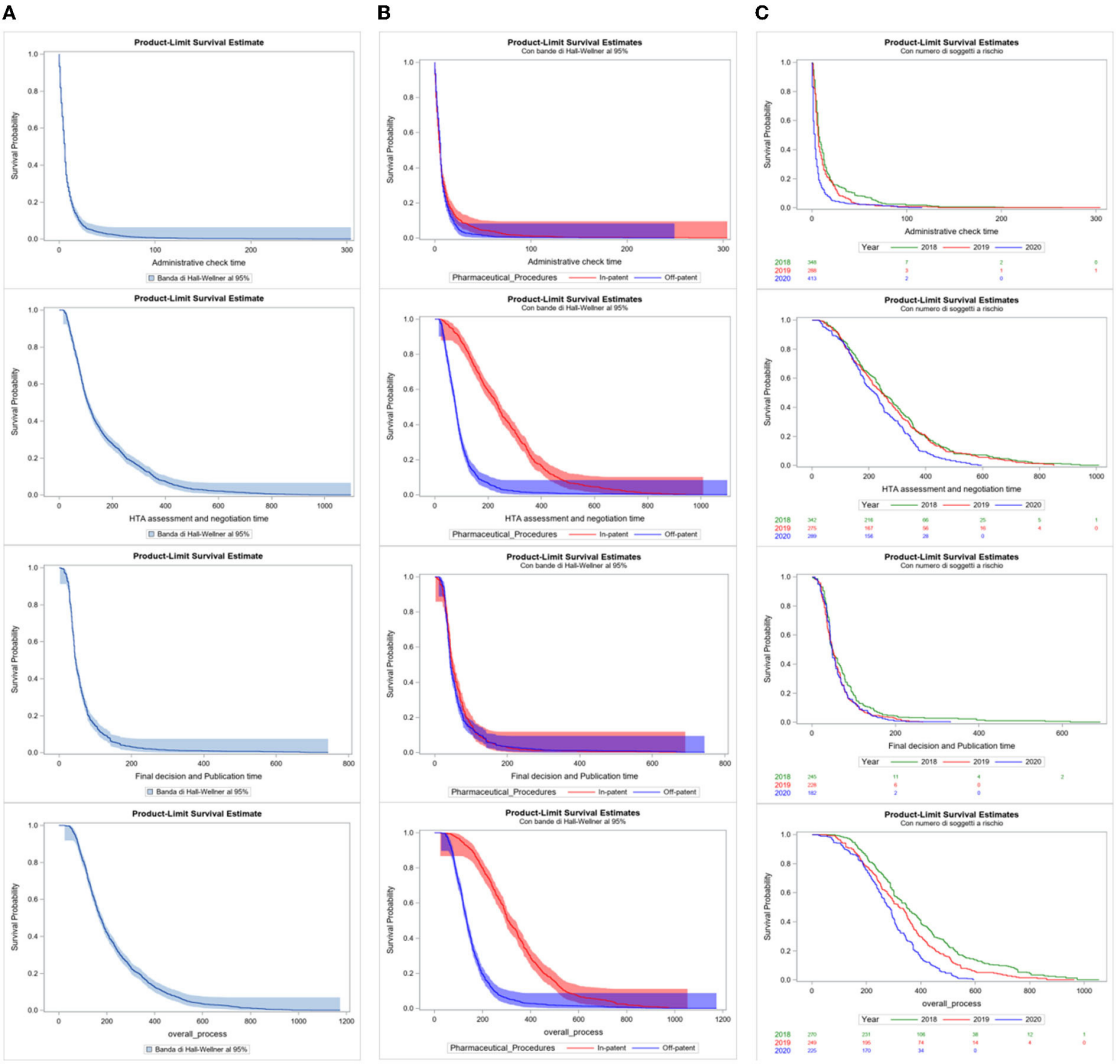
**(A–C)** Kaplan–Meier curves of time days indicators by total, by pharmaceutical procedures, and per year.

The survival analysis shows the survival probability of procedures in the period 2018–2020 based on the processing time. Figure 5 relates to the probability of survival of a procedure and the time in days of each indicator. Observing the first column of curves **(A)**, 75% of procedures have an estimated indicator value equal to 11.0 days for Administrative check time, 223.0 days for HTA assessment and negotiation time, 70.0 days for final decision and publication time, and 291.0 days for Overall process time indicator. The values of indicators 1 and 3 are confirmed around the average values between Off-patent and In-patent procedures in the period 2018–2020, while for indicators 2 and 4, the high value is more influenced by the In-patent typology of negotiation, as previously observed.

In the second column of survival curves **(B)**, the major difference in the curves between “Off-patent” (blue) and “In-patent” (red) procedures is significant by the Log-Rank Test for the second and fourth indicators (*p* < 0.0001). This confirms the significant difference in behaviors between the two partitions in the HTA assessment and negotiation and overall process time indicators: In-patent procedures take more processing time at this stage of the negotiation process. The other two curves of indicators 1 and 3 are quite overlapping.

In the third column of the survival curves of only In-patent procedures **(C)**, the presence of the differentiation of the years in the curves is statistically significant for the purposes of the negotiation process only for the HTA assessment and negotiation and overall process time indicators (*p* < 0.0001). The curves are quite close to the value in days of the median; from that moment on, they begin to diversify and 2020 is ahead of the times compared to the curves of 2018 and 2019.

In summary, indicator 2 is the most complex and longest over time, as it refers to a greater number of activities and actors involved than the other indicators and this is what influences the overall process time indicator 4 the most.

## 4. Discussion

This is the first study to examine the time of the P&R process in Italy, from MAH submission to publication of the final decision in the Italian Official Journal. The time span considered was 3 years during the first year of the COVID-19 pandemic.

The analysis, conducted from 2018 to 2020, shows that the number of procedures submitted to the HTA and Pharmaceutical Economy Division by pharmaceutical companies for the request for pricing and reimbursement is to be considered constant over time, with an average annual number of procedures equal to 815.

The highest frequency, calculated taking into account all negotiation typologies and procedures from 2018 to 2020, is attributable to Off-patent drugs, copies, and parallel imports (about 57%).

Procedures related to orphan drugs in the years 2018–2020 show a stable trend year on year, while those relating to the extension of indications, price renegotiations, and new active ingredient(s) record an increase.

In 2020, the overall process was evaluated in 129.8 average days (108.0 median value) for Off-patent pharmaceutical procedures and in 283.1 average days (284.0 median value) for In-patent pharmaceutical procedures. The overall duration of the P&R process showed a decreasing trend. There is a statistically significant difference between In-patent and Off-patent procedures, where In-patent procedures are more variable and need more time for processing.

The procedures related to new combinations of active ingredients, extensions, new active ingredient(s), orphan medicines, and renegotiations are characterized by more complexity, requiring more time to be assessed and negotiated.

The “HTA assessment and negotiation time” indicator is the step of the process that mainly influences the entire duration of the P&R process, given the complexity and the various active roles involved. For this indicator, the time needed is below 100 days for the processing of Off-patent procedures in the 3 years analyzed, whereas the overall P&R period is above 100 days for both Off-patent and In-patent procedures.

Compared to the European average times, in Italy, the times necessary for evaluation, authorization for reimbursement, and definition of the price of medicine are to be considered satisfactory. Indeed, from the W.A.I.T Patient survey (REF), it emerges that Italy ranks among the first European countries in terms of the number of medicines made accessible to patients, reimbursement, and availability times for patients.

More specifically, Italy ranks fourth in terms of the number of reimbursable medicines (114, equal to 74% of the 152 medicines authorized by EMA in the 4-year period 2016–2019 and examined in the analysis), preceded by Germany (133), Denmark (131), Austria (124), and Switzerland (115), and against an EU average of 74 drugs, corresponding to 49%.

In terms of available time, that is, the time elapsing between the marketing authorization and the access date for patients (which, in most European countries, corresponds to the time when medicines enter the reimbursement list), Italy records an average value of 418 days, after Germany (120 days), Switzerland (166 days), Denmark (169 days), The Netherlands (213 days), Sweden (262 days), Austria (302 days), England (335 days), Russia (384 days), and Macedonia (397 days), against a European average of 504 days. The times indicated by WAIT are different from those of AIFA since the indicators and the study sample are built differently. WAIT is based on innovative pharmaceuticals and orphan drugs and times are calculated from the granting of the marketing authorization to the moment when the medicines become accessible to the patient. Conversely, AIFA takes into account all negotiation typologies (orphan medicines, new chemical entities, generics, etc.) and considers the time from submission of a marketing authorization application by the MAH to the publication of the final decision in the Italian Official Journal. To some extent, the difference between WAIT and AIFA may be attributable to the waiting time between the favorable opinion issued by the CHMP and the opinion of the European Commission, which normally takes around 60 days, and the time needed by the pharmaceutical company to submit the P&R application to AIFA.

The time needed to guarantee reimbursement by the Italian NHS is slightly shorter in comparison with the European average, although a greater number of medicines are reimbursed in Italy. In addition, with regard to generics, AIFA reduced the negotiation times by introducing simplified procedures in 2021. Other simplified procedures were also introduced (e.g., parallel import procedures) and a further improvement in the timing is expected.

The analysis encompasses 2 years of the COVID-19 pandemic, during which worldwide regulatory agencies and HTA bodies have been called upon to manage the pharmaceutical needs deriving from the health emergency. AIFA and its committees worked intensively to guarantee that their ordinary activities were not severely impacted by the management of the emergency, and to ensure timely access to COVID-19 treatments and vaccines for the prevention of SARS-CoV-2 infection. For example, clinical studies were authorized for collecting rigorous data about therapies used for COVID-19, in order to provide standardized information and procedures to health professionals, thus ensuring national homogeneity. With the aim of guaranteeing these tasks, and without delaying access to all other medicines for other health needs, several measures have been implemented, including the management of P&R procedures through electronic platforms and extraordinary meetings of AIFA Committees.

In the European context, health systems and procedures to ensure access to medicines are profoundly different. For example, Germany is one of the fastest countries in terms of access time to drugs, with a health system that provides for immediate reimbursement once the pharmaceutical is authorized. Additionally, the cost–benefit assessment is performed at a later stage, usually 2 years later. In England, access to medicines is made immediately available after the authorization phase, and National Institute for Health and Care Excellence (NICE) provides HTA recommendations, when conducted, at a later stage (15).

In Italy, the reimbursement of medicine occurs only after the clinical and economic evaluation and the price negotiation. This approach makes it possible to guarantee full reimbursement according to the value of the treatment. Furthermore, in order to guarantee the fastest access to promising therapies and to conduct negotiation procedures more easily, various early access programs are in place, intended to ensure access before the Marketing Authorization is granted or before the medicine is made available following the decision on reimbursement and price negotiation. These programs are financed by the NHS (Law 648/96) or through dedicated resources, funded by Industry, and AIFA National Fund (Law 326/2003-−5% Fund). In addition, since 2012, medicines authorized through the centralized procedure, pursuant to Regulation 726/2004, have been placed in class CNN within 60 days of EC approval, providing immediate access to patients, although not reimbursed or at hospital expenses. In this case (refer to Supplementary material), the negotiation of the reimbursed price takes place subsequently, at the request of the MAH. Therefore, pending the conclusion of the negotiation procedure, such drugs may be made available to patients beforehand, as they can be purchased directly by the health authorities (e.g., Hospital/Local Health Authority). Conversely, for new indications, given that the pharmaceutical is already reimbursed and on the market, it can be made immediately available.

### 4.1. Limitations

The analysis conducted has some limitations. These are mainly due to the different completion stages of the procedures submitted in the 3-year period considered, which also produce their effects in terms of comparability of the results per year, especially for 2020, where data relating to such procedures were still incomplete. These effects will be addressed in subsequent analyses with a new update.

The analysis has been conducted exclusively in the national context and without considering other useful indicators and has taken into account the time from the CHMP opinion and/or the EC Decision to the submission of a P&R application by the MAHs.

No quality indicators have been developed to evaluate the complexity of the entire duration of the P&R process, and this aspect could be analyzed in a second future analysis.

As a further limitation, the entire process does not distinguish the potential clock-stops of the negotiation procedure, which are due to the request for additional information and delayed submissions with respect to the scheduled Committee meeting, as well as suspensions requested by the company for the formulation of the new proposal. In particular, the clock-stops significantly affect the timing of the analysis by expanding its value compared to the actual number of days required to complete the Price and Reimbursement procedure.

## Author contributions

VG, EA, GA, and FT: conception and study design. EA: collection, assembly of data, and performed the data analysis. SM, VG, and EA: analysis, interpretation, and manuscript writing. FT: methodological advice. All authors read, revised, and approved the final manuscript.
