# Supplementary material for: The timelines for the price and reimbursement authorization in Italy 2018–2020
Source: Front Med (Lausanne). 2022 Dec 21;9:1055359. doi: 10.3389/fmed.2022.1055359 (PMC9810802; doi:10.3389/fmed.2022.1055359)
Supplement: Supplementary file 1 [file Data_Sheet_1.pdf]

Supplementary Material

Number of words: 525

Number of tables: 2

1 The reimbursement system

The Italian pharmaceutical reimbursement system covers all relevant diseases and the entire country providing universal pharmaceutical coverage to the whole population, including legal residents, and fully covering both prescription medicines for primary care and medicines for in-patient care.

The current reimbursement classification groups medicines into the following reimbursement classes, according to a combination of criteria in terms of effectiveness and cost:

- Class A, comprising essential medicines and medicines for serious and chronic diseases. Medicines of this class are fully reimbursed by NHS.
- Class H, consisting of medicines requiring specialist's supervision, are eligible for reimbursement only when used for in-patient care (hospital use only - or HOM).
- Class C, consisting of medicines not reimbursed by the NHS: pharmaceutical for disease of slight importance and for minor ailments, or medicines whose use is discouraged or without proven efficacy, and medicines not requiring a medical prescription; furthermore in this class medicines who broke up negotiation are included. Non-reimbursable pharmaceuticals include over-the-counter (OTC) products and non-prescription pharmaceuticals (SOP) (a subgroup of OTC products).
- Class C-NN (C non negotiated), including pharmaceuticals whose prices have not been negotiated. The law no. 189/2012 established that medicinal products, which are granted a marketing authorization through centralised, mutual recognition, decentralised or national procedure, as well as through parallel import, are automatically classified in the class C-NN, pending the submission, by the marketing authorisation holder (MAH), of an application for a different classification and for price negotiation, following submission of a specific dossier, according to CIPE Resolution (see infra). Before marketing, the MAH is required to communicate to AIFA the ex factory price and the retail price of the medicinal product classified in class C-NN, together with the marketing date.

In Italy, as of 1st January 2004, the price setting of medicinal products reimbursed by the National Health Service (NHS) is regulated at central level by the Italian Medicines Agency (AIFA), through negotiation procedures with the pharmaceutical companies. Prices of non-reimbursed medicines are, instead, freely established, with some limitations, by MAHs.

2 Supplementary Data

In this supplementary section, the details of the pharmaceuticals procedures are shown for the entire period from 2018 to 2020, and stratified for the Anatomical Therapeutic Chemical (ATC) classification at first level and for typology of negotiation. These latter are grouped in four most important categories: New active ingredient(s) and Orphans medicines ("NAI(s)/Orphans medicines"), Extension of therapeutic indications / posology, Off-patent pharmaceuticals procedures (generics, biosimilars, copies and/or parallel trade) and Other (where all the others are).

In the period, a greater number of procedures pertains to medicines classified as L-Antineoplastic and immunomodulating agents (462, 18.9%) and as N-Nervous system (348, 14.2%), followed by medicines of A-Alimentary Tract and Metabolism (308, 12.6%) and of C-Cardiovascular system (307, 12.6%) groups. The same trend is observed in each year of the period.

From Table 1 it is observed how the concentration of the typologies of indication of the I level of ATC stands between the extensions, off-patents and others. The first level A from 2018 there is a sharp decline in off-patent.

Supplementary Table 1: Procedures distribution for each first level of ATC by typology of negotiation per year.

	2018		2019		2020		2018-2020	
**I level of ATC / Typology of negotiation**	**n**	**%**	**n**	**%**	**n**	**%**	**n**	**% (desc. ord.)**
**L**	**136**	**14.5%**	**141**	**20.8%**	**185**	**22.3%**	**462**	**18.9%**
NAI(s)/Orphans medicines	15	11.0%	15	10.6%	22	11.9%	52	11.3%
Extension of therapeutic indications / posology	43	31.6%	45	31.9%	58	31.4%	146	31.6%
Off-patent (generics. biosimilars. copies and/or parallel trade)	42	30.9%	43	30.5%	41	22.2%	126	27.3%
Other	36	26.5%	38	27.0%	64	34.6%	138	29.9%
**N**	**127**	**13.6%**	**105**	**15.5%**	**116**	**14.0%**	**348**	**14.2%**
NAI(s)/Orphans medicines	3	2.4%	9	8.6%	4	3.4%	16	4.6%
Extension of therapeutic indications / posology	6	4.7%	0	0.0%	10	8.6%	16	4.6%
Off-patent (generics. biosimilars. copies and/or parallel trade)	103	81.1%	77	73.3%	85	73.3%	265	76.1%
Other	15	11.8%	19	18.1%	17	14.7%	51	14.7%

	2018		2019		2020		2018-2020	
**I level of ATC / Typology of negotiation**	**n**	**%**	**n**	**%**	**n**	**%**	**n**	**% (desc. ord.)**
**A**	**166**	**17.7%**	**60**	**8.9%**	**82**	**9.9%**	**308**	**12.6%**
NAI(s)/Orphans medicines	10	6.0%	4	6.7%	4	4.9%	18	5.8%
Extension of therapeutic indications / posology	8	4.8%	6	10.0%	9	11.0%	23	7.5%
Off-patent (generics. biosimilars. copies and/or parallel trade)	113	68.1%	33	55.0%	45	54.9%	191	62.0%
Other	35	21.1%	17	28.3%	24	29.3%	76	24.7%
**C**	**157**	**16.8%**	**71**	**10.5%**	**79**	**9.5%**	**307**	**12.6%**
NAI(s)/Orphans medicines	1	0.6%	1	1.4%	4	5.1%	6	2.0%
Extension of therapeutic indications / posology	15	9.6%	2	2.8%	11	13.9%	28	9.1%
Off-patent (generics. biosimilars. copies and/or parallel trade)	88	56.1%	41	57.7%	32	40.5%	161	52.4%
Other	53	33.8%	27	38.0%	32	40.5%	112	36.5%
**G**	**71**	**7.6%**	**76**	**11.2%**	**70**	**8.4%**	**217**	**8.9%**
NAI(s)/Orphans medicines	1	1.4%	1	1.3%	1	1.4%	3	1.4%
Extension of therapeutic indications / posology	0	0.0%	0	0.0%	0	0.0%	0	0.0%
Off-patent (generics. biosimilars. copies and/or parallel trade)	60	84.5%	64	84.2%	57	81.4%	181	83.4%
Other	10	14.1%	11	14.5%	12	17.1%	33	15.2%
**J**	**75**	**8.0%**	**64**	**9.5%**	**74**	**8.9%**	**213**	**8.7%**
NAI(s)/Orphans medicines	3	4.0%	6	9.4%	10	13.5%	19	8.9%
Extension of therapeutic indications / posology	9	12.0%	13	20.3%	12	16.2%	34	16.0%
Off-patent (generics. biosimilars. copies and/or parallel trade)	50	66.7%	36	56.3%	27	36.5%	113	53.1%
Other	13	17.3%	9	14.1%	25	33.8%	47	22.1%

I level of ATC / Typology of negotiation	2018		2019		2020		2018-2020	
	n	%	n	%	n	%	n	% (desc. ord.)
**B**	**46**	**4.9%**	**34**	**5.0%**	**37**	**4.5%**	**117**	**4.8%**
NAI(s)/Orphans medicines	5	10.9%	6	17.6%	5	13.5%	16	13.7%
Extension of therapeutic indications / posology	5	10.9%	1	2.9%	1	2.7%	7	6.0%
Off-patent (generics. biosimilars. copies and/or parallel trade)	25	54.3%	12	35.3%	15	40.5%	52	44.4%
Other	11	23.9%	15	44.1%	16	43.2%	42	35.9%
**M**	**49**	**5.2%**	**32**	**4.7%**	**29**	**3.5%**	**110**	**4.5%**
NAI(s)/Orphans medicines	1	2.0%	1	3.1%	2	6.9%	4	3.6%
Extension of therapeutic indications / posology	3	6.1%	1	3.1%	3	10.3%	7	6.4%
Off-patent (generics. biosimilars. copies and/or parallel trade)	41	83.7%	24	75.0%	20	69.0%	85	77.3%
Other	4	8.2%	6	18.8%	4	13.8%	14	12.7%
**S**	**35**	**3.7%**	**22**	**3.2%**	**55**	**6.6%**	**112**	**4.6%**
NAI(s)/Orphans medicines	2	5.7%	1	4.5%	1	1.8%	4	3.6%
Extension of therapeutic indications / posology	1	2.9%	1	4.5%	0	0.0%	2	1.8%
Off-patent (generics. biosimilars. copies and/or parallel trade)	27	77.1%	16	72.7%	47	85.5%	90	80.4%
Other	5	14.3%	4	18.2%	7	12.7%	16	14.3%
**R**	**30**	**3.2%**	**24**	**3.5%**	**49**	**5.9%**	**103**	**4.2%**
NAI(s)/Orphans medicines	1	3.3%	1	4.2%	1	2.0%	3	2.9%
Extension of therapeutic indications / posology	2	6.7%	6	25.0%	7	14.3%	15	14.6%
Off-patent (generics. biosimilars. copies and/or parallel trade)	20	66.7%	14	58.3%	25	51.0%	59	57.3%
Other	7	23.3%	3	12.5%	16	32.7%	26	25.2%

	2018		2019		2020		2018-2020	
**I level of ATC / Typology of negotiation**	**n**	**%**	**n**	**%**	**n**	**%**	**n**	**% (desc. ord.)**
**H**	**12**	**1.3%**	**25**	**3.7%**	**20**	**2.4%**	**57**	**2.3%**
NAI(s)/Orphans medicines	1	8.3%	1	4.0%	1	5.0%	3	5.3%
Extension of therapeutic indications / posology	2	16.7%	0	0.0%	2	10.0%	4	7.0%
Off-patent (generics. biosimilars. copies and/or parallel trade)	6	50.0%	20	80.0%	8	40.0%	34	59.6%
Other	3	25.0%	4	16.0%	9	45.0%	16	28.1%
**D**	**15**	**1.6%**	**12**	**1.8%**	**16**	**1.9%**	**43**	**1.8%**
NAI(s)/Orphans medicines	2	13.3%	0	0.0%	1	6.3%	3	7.0%
Extension of therapeutic indications / posology	0	0.0%	4	33.3%	1	6.3%	5	11.6%
Off-patent (generics. biosimilars. copies and/or parallel trade)	9	60.0%	4	33.3%	8	50.0%	21	48.8%
Other	4	26.7%	4	33.3%	6	37.5%	14	32.6%
**V**	**18**	**1.9%**	**9**	**1.3%**	**16**	**1.9%**	**43**	**1.8%**
NAI(s)/Orphans medicines	3	16.7%	1	11.1%	2	12.5%	6	14.0%
Extension of therapeutic indications / posology	2	11.1%	1	11.1%	0	0.0%	3	7.0%
Off-patent (generics. biosimilars. copies and/or parallel trade)	5	27.8%	3	33.3%	5	31.3%	13	30.2%
Other	8	44.4%	4	44.4%	9	56.3%	21	48.8%
**P**	**0**	**0.0%**	**2**	**0.3%**	**3**	**0.4%**	**5**	**0.2%**
NAI(s)/Orphans medicines	0	0.0%	0	0.0%	0	0.0%	0	0.0%
Extension of therapeutic indications / posology	0	0.0%	0	0.0%	0	0.0%	0	0.0%
Off-patent (generics. biosimilars. copies and/or parallel trade)	0	0.0%	2	100.0%	3	100.0 %	5	100.0%
Other	0	0.0%	0	0.0%	0	0.0%	0	0.0%

I level of ATC / Typology of negotiation	2018		2019		2020		2018-2020	
	n	%	n	%	n	%	n	% (desc. ord.)
**Total**	**937**	**100.0 %**	**677**	**100.0 %**	**831**	**100.0 %**	**2445**	**100.0 %**

Legend: A-Alimentary tract and metabolism; B-Blood and blood forming organs; C-Cardiovascular system; D-Dermatologicals; G-Genito-urinary system and sex hormones; H-systemic hormonal preparations. excluding sex hormones and insulins; J-Antiinfectives for systemic use; L-Antineoplastic and immunomodulating agents; M-Musculo-skeletal system; N-Nervous system; P-Antiparasitic products. insecticides and repellents; R-Respiratory system; S-Sensory organs; V-Various

In order to better clarify the specific time indicators relevant to extensions of indication, new active substances and orphan products, Table 2 is shown below.

Supplementary Table 2: Time in days indicators in 2020. stratifying by typology of negotiation procedure.

Time indicators	Period	% procedures completed	N	Mean	95% CI (inf-sup)	Median	Q1	Q3	IQR (Q3-Q1)	Range (min-max)
Active principle associations	2020	603/831 (73%)	4	305	(201.3-408.7)	277	239	372	610	(210-456)
Dosage unit variation			16	294	(235.1-352.4)	290	198	363	561	(116-570)
Extensions of indication			43	293	(259.1-327.1)	293	210	371	581	(24-593)
Generics. biosimilars. copies. paralell trade			378	130	(122.3-137.2)	108	79	162	241	(28-504)
New active ingredient(s)			22	337	(280.6-392.6)	356	273	430	703	(66-527)
Orphan medicines			7	327	(248-406.3)	342	266	390	656	(150-489)
Other (e.g. market shortages)			4	238	(202.2-272.8)	250	215	261	475	(185-265)
Reimbursement reclassification			5	111	(64.1-158.7)	72	72	169	241	(72-172)
Renegotiations			48	316	(283.2-348.1)	302	241	374	614	(31-589)
Variation of packages			76	248	(223.9-271.6)	245	187	312	499	(57-520)
